# Prediction of Adverse Perinatal Outcome in Monochorionic Twin Pregnancy Using Fetal Biometry and Doppler Data: A Multicentre Cohort Study

**DOI:** 10.1111/1471-0528.70116

**Published:** 2025-12-16

**Authors:** Miriam Lopian, Veronica Giorgione, Mariarita Trapani, Mariafrancesca Brutto, Maria Giulia Ferrante, Amar Bhide, Jacques C. Jani, Dominique A. Badr, Tullio Ghi, Elisa Bevilacqua, Basky Thilaganathan, Alessandra Familiari

**Affiliations:** ^1^ Fetal Medicine Unit St George's University Hospitals NHS Foundation Trust London UK; ^2^ Department of Women and Child Health, Women Health Area Fondazione Policlinico Universitario Agostino Gemelli IRCCS Rome Italy; ^3^ Vascular Biology Research Centre, Molecular and Clinical Sciences Research Institute St George's University of London London UK; ^4^ Department of Obstetrics and Gynecology, University Hospital Brugmann Université Libre de Bruxelles Brussels Belgium; ^5^ Catholic University of the Sacred Heart Rome Italy

**Keywords:** adverse perinatal outcome, Doppler, estimated fetal weight, fetal weight discordance, monochorionic, selective fetal growth restriction, stillbirths, twin pregnancies

## Abstract

**Objective:**

To assess whether combining estimated fetal weight (EFW) and fetal Doppler ultrasound parameters would provide information to optimize the timing of birth in monochorionic twin pregnancies and prevent adverse perinatal outcomes.

**Study Design:**

Retrospective multicentre cohort study.

**Setting:**

Three tertiary centres in the UK, Italy and Belgium.

**Population:**

624 monochorionic twin pregnancies managed between 2013 and 2023.

**Methods:**

Univariable and multivariable analyses assessed the association between EFW and Doppler indices taken within 2 weeks of birth.

**Main Outcome Measures:**

Stillbirth at any gestation or iatrogenic preterm birth before 34 weeks for suspected fetal compromise.

**Results:**

The primary outcome occurred in 143 (22.9%) pregnancies with 70 cases of early PTB for fetal reasons and 73 cases of at least one IUD. Significant associations between biometric and Doppler parameters and adverse perinatal outcomes were found. The best‐performing prediction models incorporated EFW discordance and umbilical artery pulsatility index (UA PI) discordance, achieving an AUC of 0.85 (95% CI 0.78–0.91) and EFW discordance and absent or reverse end diastolic flow of UA PI with an AUC of 0.86 (95% CI 0.80–0.92). The model incorporating EFW and UA PI discordance could be applied to the largest proportion of pregnancies and outperformed the currently clinical sFGR classification in predicting adverse outcomes.

**Conclusion:**

A model incorporating intertwin EFW discordance and UA PI discordance outperforms the current clinical classification for prediction of adverse perinatal outcomes in monochorionic pregnancies. If confirmed by further external validation studies, these findings could contribute to building a tailored risk assessment in these pregnancies.

## 1 Introduction

Twin pregnancies are at increased risk of adverse perinatal outcomes compared to singletons, and monochorionic twins, in particular, are burdened by the highest risk of adverse outcomes [1]. Indeed, alongside the risks of early preterm birth and placental insufficiency that are common among all twins, monochorionic pregnancies are susceptible to additional complications owing to their shared placenta and the consequent shared fetal‐fetal circulation [2, 3].

Selective fetal growth restriction (sFGR) occurs because of unequal placental sharing and imbalances in vascular anastomoses, resulting in growth discordance between foetuses that are almost always considered to have an identical genetic growth potential [3, 4, 5, 6]. The management of pregnancies complicated by sFGR aims to strike a delicate balance between two primary objectives: prolonging the pregnancy to reduce complications for both twins associated with iatrogenic preterm birth and mitigating the risk of fetal death in the growth‐restricted twin because of worsening discordant placental dysfunction [7]. Identifying foetuses at high risk of Intrauterine Death (IUD) is particularly important in these pregnancies because the demise of one twin places the other co‐twin at risk of IUD or neurodevelopmental impairment [8].

The current classification of sFGR provides a useful risk stratification based on the diastolic flow pattern in the Umbilical Artery (UA) of the growth‐restricted twin [9]. Type I exhibits persistent positive end‐diastolic flow (EDF) and is associated with the most favorable prognosis, while persistent absent or reversed end‐diastolic flow (AREDF) characterizes Type II. Type III with intermittent AREDF has been shown to be associated with the poorest prognosis, resulting in IUD or parenchymal brain damage in up to 20% of cases [10]. Despite being developed on a case series of only 134 sFGR pregnancies involving 16 IUDs, this classification system has been widely adopted into clinical practice. However, this classification system is solely reliant on UA Doppler pattern of the smaller twin without considering other critical prognostic factors that are known to be informative in the management of singleton pregnancies [11, 12, 13, 14].

Currently, there is no clinical classification system that quantifies the risks of adverse pregnancy outcomes with sFGR, accounting for the complex interaction of gestational age, fetal weight discordance and other Doppler parameters. This underscores the urgent need for more robust, data‐driven decision‐making algorithms or tools to optimise outcomes in these complex pregnancies. This multicentre study aims to develop a prediction model for adverse outcomes in monochorionic twin pregnancies, incorporating gestational age, intertwin discordance, estimated fetal weight (EFW) and Doppler parameters, addressing key limitations of the existing classification system.

## 2 Methods

### 2.1 Study Design

This study was a retrospective, multicentre cohort study conducted at three tertiary medical centres across Europe, all referral centres for twin pregnancy management and fetal intervention. The participating institutions were St George's Hospital in London, United Kingdom; Fondazione Policlinico Universitario Agostino Gemelli IRCCS in Rome, Italy; and University Hospital Brugmann in Brussels, Belgium. This manuscript follows the TRIPOD guidelines to ensure clear, thorough, and transparent reporting of the development and validation of the prediction model [15]. The study included patients with monochorionic twin pregnancies managed at these centres between 2013 and 2023.

### 2.2 Study Participants

Patients with monochorionic diamniotic twin pregnancies (MCDA) were included if they underwent an obstetric ultrasound evaluation within 2 weeks of the birth or of the outcome of interest (IUD or iatrogenic preterm delivery < 34 weeks of gestation for suspected fetal compromise). Exclusion criteria included pregnancies complicated by structural anomalies, chromosomal abnormalities, miscarriage (up to 15 + 6 weeks), monochorionic monoamniotic, and dichorionic twin pregnancies. Gestational age and chorionicity were determined through an ultrasound examination performed between 11 and 14 weeks of gestation. Chorionicity was determined by the evaluation of the intertwin membrane at its insertion into the placenta. The “lambda sign” was observed in dichorionic twins, whereas the “T sign” was characteristic of monochorionic twins [16]. Each participating medical centre obtained the requisite ethical approvals or waivers for retrospective data analysis from their respective institutional review boards or ethics committees.

### 2.3 Data Acquisition

Data regarding maternal demographics, pregnancy characteristics, sonographic parameters as well as obstetric and neonatal outcomes were collected from the electronic patient database. For consistency in labeling during the analysis of the data, the smaller twin was designated as Twin 1 and Twin 2 as the larger twin, based on the last recorded EFW prior to delivery, with this designation confirmed by birthweight after delivery. The last recorded EFW for each twin was calculated using the Hadlock‐3 formula [17]. Gestational age‐specific EFW centiles were then determined based on the Fetal Medicine Foundation charts for singletons [18]. The CPR was calculated as the simple ratio between the MCA PI and the UA PI [14]. EFW discordance was calculated using the following formula: (EFW larger twin−EFW smaller twin)/EFW larger twin*100. Small for gestational age (SGA) was defined as EFW below the 10th or 3rd centile for gestational age. The intertwin discordance for UA and MCA pulsatility indices (PI) was determined using their last recorded values. Discordance was calculated as a percentage: (Higher PI − Lower PI)/Higher PI × 100. Pregnancies with sFGR were categorized using the Gratacos classification system based on UA Doppler parameters assessed prior to delivery, as it is the one most widely used currently for risk stratification and clinical management [9].

### 2.4 Outcomes

The main outcome of the study was a composite of: IUD of at least one foetus at any gestation or suspected fetal compromise necessitating iatrogenic early preterm birth (PTB) (< 34 weeks). Suspected fetal compromise was determined using specific Doppler and cardiotocography (CTG) parameters. Doppler criteria included the presence of persistent or intermittent absent or reversed end‐diastolic flow (AEDF/REDF) in the umbilical artery after 30–32 weeks, and the detection of an absent or reversed a‐wave in the ductus venosus at any gestational age. CTG criteria for delivery were defined as recurrent or persistent unprovoked decelerations, or short‐term variability (STV) less than 2.5 ms before 28 weeks, less than 3.0 ms between 28 and 32 weeks, or less than 3.5 ms before 34 weeks. Cases of early PTB that were either spontaneous or iatrogenic for indications other than suspected fetal compromise (i.e., preeclampsia or rupture of membranes) were not included in the primary outcome. IUD was defined as the demise of at least one foetus from 16 weeks of gestation to capture clinically significant losses related to monochorionic‐specific complications, which typically manifest between 16 and 24 weeks. This definition aligns with clinical practice, where intensive surveillance and potential interventions for monochorionic twins are initiated from as early as 16 weeks, reflecting the critical period for these high‐risk pregnancies [16].

### 2.5 Statistical Analysis

The study's sample size was defined by the number of eligible cases present in the datasets from the three participating centres, rather than being established through formal sample size calculations. Data from categorical variables was expressed as *N* (%) and from continuous variables as median and interquartile range (IQR). Logistic regression models were used to evaluate the association between various ultrasound parameters (EFW, UA and MCA discordance) and adverse perinatal outcomes. Multivariable models were also performed to adjust for confounders including maternal age, non‐white ethnicity, BMI and gestational age at ultrasound. Receiver Operating Characteristic (ROC) curves were constructed to demonstrate the predictive accuracy of various variables and combinations of variables at predicting adverse perinatal outcomes in pregnancies after exclusion of Twin‐to‐Twin Transfusion Syndrome (TTTS) cases. The results were reported as the area under the curve (AUC) and 95% confidence interval (95% CI). TTTS cases were diagnosed according to the Quintero staging criteria [19] and were excluded from the main analysis, as this would represent the typical clinical scenario at gestations beyond 24 weeks, where decisions about scheduled birth for sFGR would be made. In line with recommendations for prediction model development in small datasets, we assessed the adequacy of our sample size using the framework proposed by Riley et al. [20]. With 57 outcome events in our dataset after excluding TTTS cases (*n* = 487), and considering an expected Cox‐Snell *R* ^2^ between 0.10 and 0.15 and a shrinkage factor of ≥ 0.9, our sample size would support a model with up to 4–5 parameters. To minimise overfitting and preserve model reliability in this limited event‐per‐variable context, we conservatively restricted our final models to a maximum of three predictors, in line with established recommendations [20]. These decisions, combined with internal validation via 1000 bootstrap resamples, support the robustness and feasibility of our modelling strategy. Internal validation was performed via bootstrap resampling (1000 iterations) applied to the final selected model, which was defined a priori based on predictive performance and clinical relevance. As the modelling strategy was prespecified and limited to a small number of key variables, bootstrapping was not repeated across the entire variable selection process. Given the low percentage of missing data for essential variables such as EFW and UA PI (< 10%), a complete‐case analysis was employed to manage missing values. Cases lacking outcome data or missing ultrasound assessments before the event of interest were excluded from the outset by the three participating centers. The variables included in the final predictive models were chosen based on their statistical and clinical significance. Multicollinearity was assessed using the Variance Inflation Factor (VIF) and tolerance values. All predictor variables exhibited low VIF values (below 5), indicating minimal multicollinearity. Correspondingly, tolerance values were high (above 0.2), further confirming the absence of significant multicollinearity among the independent variables. Calibration of the predicted probabilities obtained using the model including EFW and UA PI discordance was evaluated using a smoothed calibration curve with 200 bootstrap resamples. A logistic regression model was fitted to the outcome, and calibration accuracy was summarised by the mean absolute error (MAE), mean squared error (MSE), and the 90th percentile of absolute error. Models were also developed in the entire MCDA cohort with TTTS cases included, and they are shown in Table S2. A sensitivity analysis was performed, including only pregnancies in which the last ultrasound examination was conducted before 34 + 0 weeks of gestation (Table S3). The screening performance of sFGR Type I, II and III was evaluated using sensitivity and specificity. The statistical analyses were performed using SPSS 28.0 (SPSS Inc., Chicago, IL, USA) and R version 4.3.2 (R Core Team, 2024) with statistical significance set at a *p*‐value of < 0.05.

## 3 Results

### 3.1 Study Cohort

**FIGURE 1:**
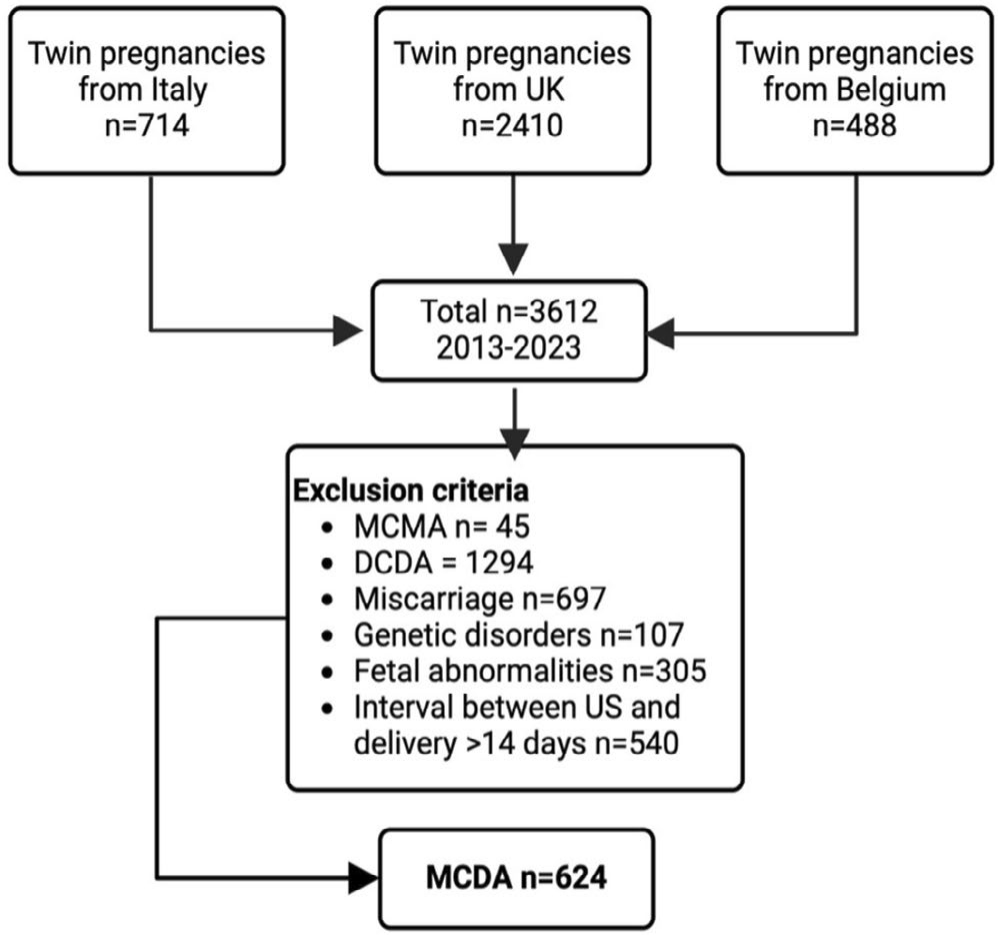
Patients flowchart. Created in https://BioRender.com. DCDA, dichorionic diamniotic; MCDA, monochorionic diamniotic; MCMA, Monochorionic monoamniotic; US, ultrasound assessment.

The final cohort included 624 MCDA pregnancies from Italy (*n* = 197), Belgium (*n* = 90), and the UK (*n* = 337, Figure 1). The maternal characteristics and perinatal outcomes of the study cohort are shown in Table 1. There were 137 (22%) pregnancies complicated by TTTS, and the rates of Type I, II, and III sFGR in our data cohort were 13.9%, 4.8% and 1%, respectively. The rate of spontaneous early PTB < 34 weeks was 86 (13.8%), with 70 (11.2%) cases being iatrogenic early PTB for fetal indications and 91 (14.6%) cases being iatrogenic early PTB for non‐fetal indications. There were 72 and 39 pregnancies affected by stillbirth in the smaller and larger twins, respectively. The primary outcome of the pre‐specified adverse perinatal outcome occurred in 143 (22.9%) pregnancies, with 70 cases of early PTB for fetal reasons and 73 cases of at least one IUD.

**TABLE 1:** Maternal demographics and fetal outcomes of monochorionic diamniotic cohort (*n* = 624).

Variables		Total pregnancies
Maternal age (years)		32 (29–36)
BMI (kg/m^2^)		24 (22–28)
Non‐white ethnicity		94/527 (17.8)
Smoking		32 (5.1)
Alcohol		13/575 (2.3)
IVF conception		36/616 (5.8)
Antenatal complications		
TTTS		137 (22)
SGA < 10th centile^a^		410 (67.8)
SGA < 3rd centile^a^		321 (53.1)
sFGR type I		87 (13.9)
sFGR type II		30 (4.8)
sFGR type III		6 (1)
Birth		
Spontaneous early PTB		86 (13.8)
Iatrogenic early PTB for non‐fetal indications		91 (14.6)
Iatrogenic early PTB for fetal indications		70 (11.2)
GA at delivery (days)		246 (223–254)
BW centile first twin smaller		1.35 (0.08–7.76)
BW centile second twin larger		14.47 (4.21–35.55)
Outcomes		
Outcome first twin (smaller)	Livebirth	533 (85.4)
	Stillbirth	72 (11.5)
	Neonatal death	18 (2.9)
Outcome second twin (larger)	Livebirth	569 (91.2)
	Stillbirth	39 (6.3)
	Neonatal death	16 (2.6)
Double loss (Stillbirth or neonatal death)		55 (8.8)
Early Intrauterine death 16–22 weeks (smaller)		38 (52.1)
Early Intrauterine death 16–22 weeks (larger)		21 (53.4)

*Note:* Data are presented as *N* (%) or median (IQR).

Abbreviations: BMI, body mass index; BW, birthweight; IVF, In vitro fertilisation; PTB, preterm birth before 34 weeks' gestation; sFGR, Selective fetal growth restriction; SGA, small for gestational age; TTTS, Twin to twin transfusion syndrome.

^a^ At least one twin (most SGA is within first twin smaller).

### 3.2 Univariate and Multivariate Analysis

Univariate analyses revealed significant associations between all examined sonographic parameters and our study outcomes. These parameters included EFW discordance and Doppler discordance (UA PI, MCA PI and CPR) as a continuous variable as well as at specific cut‐offs (Table 2). Pregnancies complicated by adverse perinatal outcomes exhibited markedly greater intertwin EFW discordance (24.5% vs. 9.1%, *p* < 0.001) and a more pronounced discordance in the PI of both the UA and MCA. Any UA PI AREDF was also significantly associated with adverse perinatal outcomes. These pregnancies also had a significantly higher incidence of TTTS (60.1% vs. 10.6%, *p* < 0.001) and SGA foetuses, both below the 10th centile (84.3% vs. 63.2%, *p* < 0.001) and below the 3rd centile (77.6% vs. 46.2%, *p* < 0.001), compared to the non‐complicated pregnancies. Multivariate logistic regression analyses demonstrated that these associations remained significant, even after adjusting for confounders such as maternal age, non‐white ethnicity, BMI and gestational age at ultrasound assessment (Table 2).

**TABLE 2:** Association between fetal factors and intrauterine death and/or iatrogenic preterm birth before 34 weeks' gestation for fetal indications.

	Livebirth (*n* = 481)	IUD or iPTB for fetal indications (*n* = 143)	OR (95% CI)	*p*	aOR^a^ (95% CI)	*p*
TTTS	51 (10.6)	86 (60.1)	12.7 (8.2–19.8)	< 0.001	2.0 (1.0–4.1)	< 0.001
EFW discordance (%)	9.06 (4.29–16.93)	24.46 (14.15–35.35)	1.1 (1.1–1.1)	< 0.001	1.1 (1.0–1.1)	< 0.001
EFW discordance > 15%	133 (28.3)	97 (72.4)	6.6 (4.3–10.2)	< 0.001	5.5 (2.8–10.9)	< 0.001
EFW discordance > 20%	92 (19.6)	77 (57.5)	5.6 (3.7–8.4)	< 0.001	4.5 (2.4–8.5)	< 0.001
EFW discordance > 25%	56 (11.9)	66 (49.3)	7.2 (4.6–11.1)	< 0.001	4.7 (2.4–9.0)	< 0.001
Any SGA < 10th centile	297 (63.2)	113 (84.3)	3.1 (1.9–5.2)	< 0.001	4.1 (2.2–7.7)^b^	< 0.001
Any SGA < 3rd centile	217 (46.2)	104 (77.6)	4.0 (2.6–6.3)	< 0.001	5.2 (3.0–8.9)^b^	< 0.001
UA PI discordance (%)	13.16 (6.41–23.33)	26.07 (9.57–44.05)	1.1 (1.0–1.1)	< 0.001	1.0 (1.0–1.1)	< 0.001
Any UA PI AREDF	8 (1.7%)	43 (32.3%)	27.9 (12.7–61.3)	< 0.001	5.4 (2.0–14.5)	< 0.001
MCA PI discordance (%)	12.40 (5.88–22.17)	21.99 (9.95–34.09)	1.0 (1.0–1.1)	< 0.001	1.0 (1.0–1.1)	< 0.001

*Note:* Data are presented as *N* (%) or median (IQR).

Abbreviations: aOR, adjusted odds ratio; AR, absent or reverse; BMI, body mass index; CI, confidence interval; EDF, end diastolic flow; EFW, estimated fetal weight; iPTB, iatrogenic preterm birth before 34 weeks' gestation; IUD, intrauterine death; MCA, middle cerebral artery; OR, odds ratio; PI, pulsatility index; SGA, small for gestational age; TTTS, twin to twin transfusion syndrome; UA, umbilical artery.

^a^ Adjusted for maternal age, BMI, non‐white ethnicity, gestational age at ultrasound.

^b^ Not adjusted for gestational age at ultrasound as centiles are already corrected for it.

### 3.3 Prediciton Modelling

Multiple prediction models for adverse perinatal outcomes were developed in monochorionic pregnancies without TTTS, incorporating various maternal and fetal parameters combinations. The predictive performance of these models using AUCs is detailed in Table 3. Models incorporating fetal parameters, including fetal growth and Doppler indices, demonstrated good predictive accuracy with AUCs ranging from 0.72 to 0.86. The best‐performing models with the fewest variables included only EFW discordance and UA PI discordance with an AUC of 0.85 (95% CI 0.78–0.91) or that with EFW discordance and any UA PI AREDF (AUC 0.86, 95% CI 0.80–0.92). The bootstrap estimates of the model including EFW and UA PI discordance confirmed the stability of the model coefficients, with minimal bias observed (bias = 0.002 for EFW discordance and 0.000 for UA PI discordance). The bias‐corrected and accelerated (BCa) 95% confidence intervals confirmed the significance of both predictors: EFW discordance (*B* = 0.097, BCa 95% CI: 0.067–0.133, *p* < 0.001) and UA PI discordance (*B* = 0.030, BCa 95% CI: 0.006–0.053, *p* = 0.008). The bootstrap‐adjusted intercept remained stable (*B* = −4.491, BCa 95% CI: −5.333 to −3.871). These findings indicate a low overfitting risk and support the robustness and generalizability of the model.

**TABLE 3:** Prediction models for intrauterine death or iatrogenic preterm birth before 34 weeks' gestation for fetal indications (*n* = 57/487 cases, after excluding cases complicated by TTTS).

Models	Variables	OR	95% CI	AUC	95% CI
*Maternal factors*					
	Maternal age (years) BMI (kg/m^2^) Non‐white ethnicity	1.00 1.06 1.01	0.93–1.04 1.00–1.12 0.46–2.20	0.61	0.52–0.69
*Fetal factors*					
1	EFW discordance (%) UA PI discordance (%) MCA PI discordance (%)	1.10 1.02 1.01	1.07–1.13 1.00–1.05 0.99–1‐04	0.82	0.75–0.90
2	EFW discordance (%) UA PI discordance (%)	1.10 1.03	1.07–1.13 1.01–1.05	0.85	0.78–0.91
3	EFW discordance (%) Any UA PI AREDF	1.11 13.60	1.08–1.14 3.16–51.18	0.86	0.80–0.92
4	SGA < 10th centile UA PI discordance (%) MCA PI discordance (%)	2.57 1.04 1.02	1.03–6.41 1.02–1.06 1.00–1.04	0.69	0.60–0.78
5	SGA < 10th centile UA PI discordance (%)	2.76 1.05	1.19–6.44 1.03–1.07	0.72	0.65–0.80
6	SGA < 10th centile Any UA PI AREDF	3.40 25.92	1.46–7.89 7.96–84.42	0.71	0.63–0.78

Abbreviations: AR, absent or reverse; AUC, area under the curve; BMI, body mass index; CI, confidence interval; EDF, end diastolic flow; EFW, estimated fetal weight; MCA, middle cerebral artery; OR, odds ratio; PI, pulsatility index; SGA, small for gestational age; UA, umbilical artery.

**FIGURE 2:**
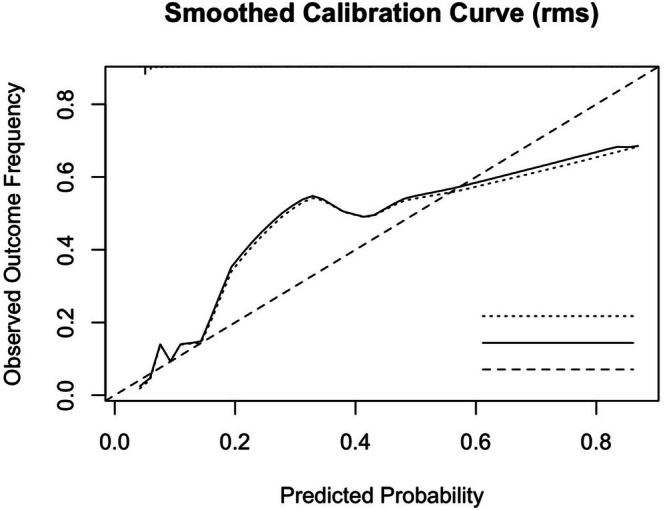
Smoothed calibration curve showing the relationship between predicted probabilities of the model including EFW and UA PI discordance and observed outcome frequencies, assessing the calibration of the predictive model. The dashed diagonal represents perfect calibration. The plot shows good agreement in the mid‐to‐high probability range, while some deviation is noted at the lower end, likely due to low event density.

Calibration of this model was assessed using a smoothed calibration curve (Figure 2). The curve demonstrated good agreement between predicted and observed risks across the mid‐to‐high probability range, with minor deviations at the lower end, with a low mean absolute error (0.033) and most deviations under 0.086. The Hosmer‐Lemeshow test was non‐significant, and model intercept and slope were close to ideal values (Table S1), further supporting adequate calibration.

Addition of MCA PI discordance to the latter model did not significantly improve performance (AUC 0.82 [95% CI 0.75–0.90]). In the sensitivity analysis restricted to pregnancies with ultrasound examinations performed before 34 weeks (*n* = 201/487; 41.3%), model performance was consistent with the main findings (Table S3). Multivariable models including TTTS performed similarly (Table S2).

**FIGURE 3:**
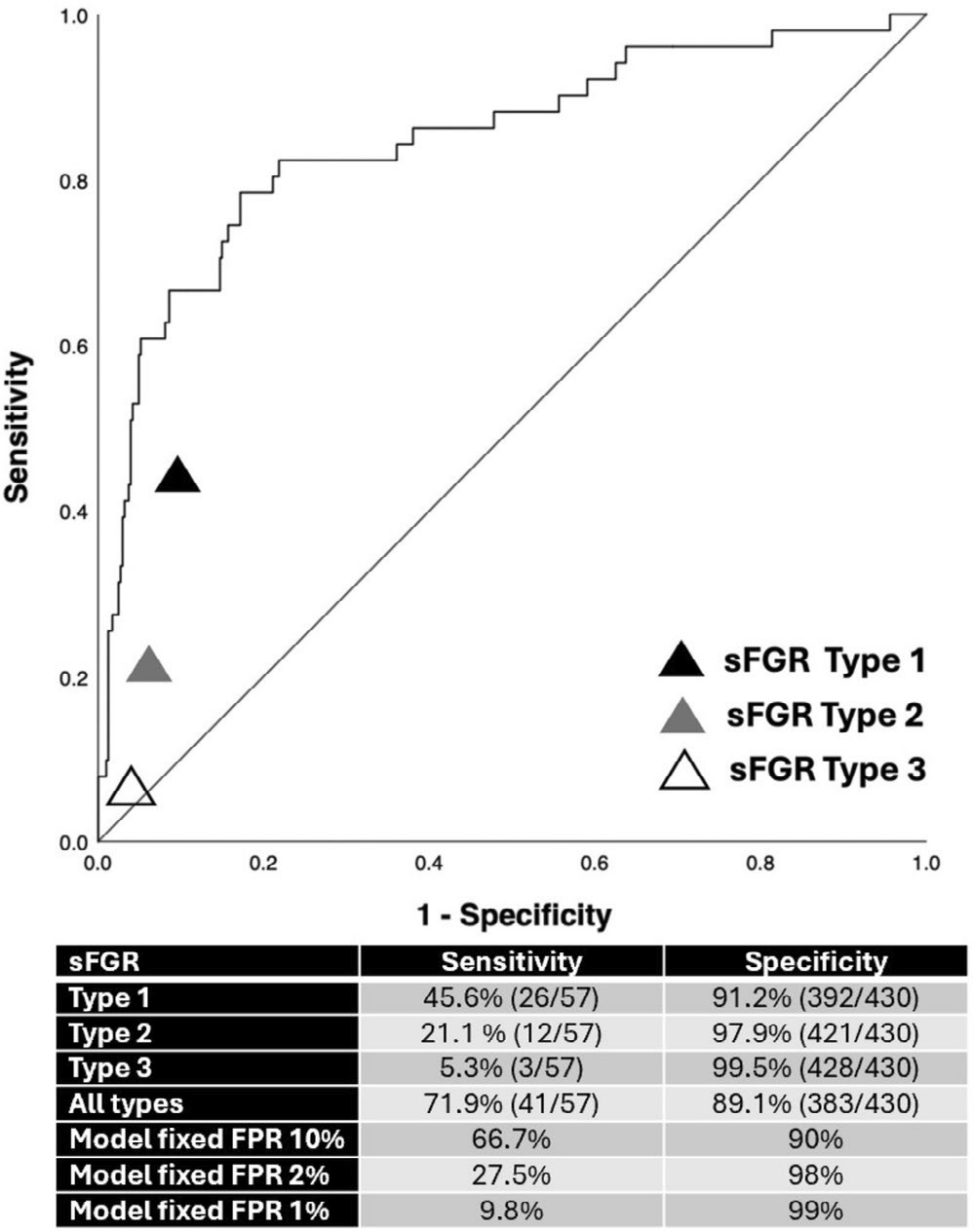
Receiver Operating Characteristic curve comparing the diagnostic performance of three different types of selective fetal growth restriction (sFGR) to our prediction model incorporating the estimated fetal weight discordance and the Umbilical Artery Pulsatility Index discordance in monochorionic twin pregnancies not complicated by twin‐to‐twin transfusion syndrome (Table 3, Model 2). The black triangle represents sFGR type 1, the grey triangle is sFGR type 2, and the white triangle is sFGR type 3 according to Gratacos classification. FPR, False positive rate; sFGR, Selective fetal growth restriction.

Figure 3 compares the predictive performance for adverse perinatal outcomes of the current classification of sFGR against one of the best‐performing models, which incorporates both EFW and UA PI discordance as continuous variables. The analysis reveals varying levels of sensitivity and specificity across the sFGR subtypes: Type I demonstrates a sensitivity of 45.6% and specificity of 91.2%, Type II shows 21.1% sensitivity and 97.9% specificity, Type III exhibits 5.3% sensitivity and 99.5% specificity, while for all sFGR types, sensitivity was 71.9% and specificity 89.1%. Overall, sFGR subtypes demonstrated low sensitivity but high specificity, with Type III demonstrating the lowest sensitivity but the highest specificity. Sensitivities for the predictive model for fixed specificities of 90%, 98% and 99% are also shown for comparison in Figure 3.

## 4 Discussion

### 4.1 Main Findings

This multicentre study demonstrates that a multimodal model incorporating EFW discordance and UA PI discordance in monochorionic pregnancies has excellent predictive accuracy for stillbirth and/or early iatrogenic PTB for suspected fetal compromise. This model outperforms the widely adopted classification of sFGR for the prediction of adverse perinatal outcomes in these pregnancies, which relies only on UA velocity pattern in pregnancies with sFGR.

### 4.2 Strengths and Limitations

The key strengths of this study include a large cohort of monochorionic twin pregnancies, being five times larger than the cohort used to develop the sFGR classification in current use. The study's primary limitation—in common with the current system in clinical use—is the retrospective design, which introduces the potential for intervention bias. In particular, the outcome of “iatrogenic preterm delivery for suspected fetal compromise*”* may introduce bias into the model, as several of the predictor variables included are likely to influence the physician's decision to proceed with elective delivery. Furthermore, despite incorporating key variables, we were unable to account for other significant predictors of adverse outcomes, such as gestational age at onset of complications and longitudinal trends in discordance. Additionally, as this is an international multicentre study conducted at tertiary referral centres, the population is represented by high‐risk cases and a bias can be assumed due to differences in clinical internal protocols. These factors may limit the generalisability of our findings to other populations and necessitate external validation before introduction to clinical practice.

### 4.3 Interpretation

The use of EFW and UA PI discordances as a continuous variable provides significantly better model performance when compared to the binary classification of SGA as EFW < 10th centile and UA PI as absent or reversed EDF. These data also highlight the superior clinical and prognostic value of inter‐twin discordance as markers of fetal well‐being in twin pregnancy, compared to arbitrary cut‐offs of EFW or Doppler parameters in either twin. It is therefore not surprising that combining these parameters in a predictive model produces excellent discrimination with AUCs > 0.8. It is meaningful to note that the high specificity of the AUC highlights the power of our model in ruling out the composite adverse outcome.

SGA defined as EFW < 10th centile is a statistical measure of size, and likely to be a less sensitive marker of pathological fetal growth restriction, as it does not include foetuses with EFW above the 10th centile but growing suboptimally relative to their co‐twin and/or genetic potential [21]. In monochorionic twins, EFW discordance may result not only from primary placental dysfunction, but also from unequal placental vascular sharing which increases the predisposition to poor growth and fetal hypoxemia [3]. The study findings are in line with the literature demonstrating that EFW discordance is a strong predictor of adverse outcomes in twins, independent of fetal size alone [22, 23]. This supports the hypothesis that the extent of fetal growth discordance rather than fetal size serves as a better proxy for the extent of underlying pathology correlating with the risk of hypoxemia and ultimately perinatal morbidity. Lastly, EFW centiles are contingent on the use of growth charts, with debate over whether singleton or twin‐specific standards better identify fetal risk [24, 25]. The current predictive model incorporated UA PI discordance, in contrast to the use of end‐diastolic velocity patterns. Elevated UA PI, a well‐established marker of placental resistance, has been shown to predict adverse outcomes in both singleton and twin pregnancies [26, 27, 28, 29]. UA PI discordance, analogous to EFW discordance, reflects inter‐twin differences in placental resistance and is a strong proxy for the underlying pathological processes that lead to adverse outcomes. The approach of continuous measurement of UA PI discordance provides a spectrum of risk assessment and a potentially more accurate reflection of underlying placental pathology and its associated perinatal risks. This approach requires that the umbilical Doppler is assessed at the same site for both twins (i.e., free cord loop close to the placenta), although it might not be easy if there is oligohydramnios of one twin.

There is a paucity of data in the literature evaluating EFW discordance and Doppler discordance as predictors of outcome in twin pregnancies, with only one previous study reporting excellent predictive performance of EFW and CPR discordance for perinatal mortality (AUC of 0.96 [95% CI 0.92–1.0]) with superior predictive performance for the CPR discordance compared to UA PI discordance [23]. In contrast, the current study finding did not show that UA and MCA PI discordance are independent risk factors for adverse outcomes. This discrepancy could be due to the inclusion of dichorionic pregnancies (75% of the cohort) in the previous study, suggesting that Doppler discordance has a greater predictive value in monochorionic twins, where the underlying primary pathology may be vascular in origin. Whereas in dichorionic twins, additional factors beyond placental sharing may contribute to adverse outcomes, potentially diminishing the relative importance of Doppler discordance [23]. The finding that MCA PI and CPR do not improve prediction is consistent with the current literature demonstrating the limited value of these parameters in predicting adverse pregnancy outcomes < 34 weeks' gestation in singleton pregnancy [30].

### 4.4 Clinical and Research Implications

The majority of national and international guidelines recommend the Gratacos classification of MCDA complicated by sFGR based on UA velocity pattern to stratify the risk for adverse outcomes and to define surveillance and management [2, 16, 31]. Generally, the guidelines further stipulate that the decision on timing of delivery should be based on the risk of fetal demise but fail to recommend methods for reliable prediction of adverse outcomes in these pregnancies. The study findings demonstrate that the combination of EFW and UA PI discordance is a better predictor of adverse pregnancy outcome compared to the current Gratacos classification system in clinical use and traditional binary cut‐offs [9]. This improved performance can be attributed not only to the use of both EFW and Doppler indices in the prediction model, but also to their use as continuous variables and to the inclusion of intertwin discordance. In contrast, the classification system in current use relies on categorical differences in UA diastolic velocity (EDF present, absent, or reversed), does not take gestational age into consideration, and is only applicable to pregnancies where one or both foetuses have an EFW < 10th centile. To the best of our knowledge, this is the first study evaluating the performance of a predictive model for adverse outcomes in monochorionic pregnancies that integrates both fetal growth and Doppler parameter discordance. Its superiority over the Gratacos classification currently endorsed by most twin pregnancy guidelines [9, 16, 31], represents a significant advancement in our understanding of growth disorders in monochorionic twin pregnancies and could fundamentally transform the monitoring and management of these pregnancies. A model that integrates EFW and UA PI discordance as continuous variables, along with gestational age, could be incorporated into clinical pathways to generate individualised probabilities of adverse outcomes following each ultrasound assessment. Pending further studies and external validation, these quantifiable probabilities would enable more precise risk stratification, supporting shared decision‐making and allowing clinicians to tailor surveillance and intervention strategies based on the calculated risk. For instance, pregnancies with increasing EFW or UA PI discordance, even if they do not reach traditional cut‐offs, could be prioritised for closer monitoring or earlier intervention, potentially reducing the risk of stillbirth and severe neonatal morbidity. Future work should focus on external validation and exploring how this model can be integrated into routine care to optimise outcomes throughout gestation.

## 5 Conclusions

This study demonstrates the value of a comprehensive approach to risk assessment in MCDA twin pregnancies. By establishing the superior predictive power of multimodal models that integrate inter‐twin discordance in both growth and Doppler parameters, our findings suggest that a shift in the evaluation of these at‐risk pregnancies could enable more precise risk stratification. Using personalised risk stratification to tailor management strategies delivers the potential to improve outcomes in these at‐risk pregnancies. Future prospective studies will be crucial in externally validating these findings and establishing their role in optimising MCDA pregnancy management protocols.

## Author Contributions

M.L., M.T., M.B. and M.G.F. collected the data from the three centres; V.G. analysed the final dataset and wrote the first draft of the manuscript; A.B., J.C.J., D.A.B., E.B. and T.G. critically reviewed the manuscript; B.T. and A.F. planned the study, interpreted the results, and reviewed the first draft of the manuscript.

## Funding

The authors did not receive any specific funding for this study.

## Ethics Statement

Our study was conducted using fully anonymised retrospective clinical data, ensuring compliance with ethical and regulatory requirements across all participating centres. In the UK, the Health Research Authority (HRA) decision tool confirmed that NHS REC review was not required, as our study did not involve identifiable patient data, interventional procedures, or the disclosure of confidential data without consent. Under UK GDPR and the Data Protection Act 2018, anonymised data is not considered personal data, and similar principles apply under EU GDPR in Italy and Belgium. In both countries, retrospective studies using fully anonymised data do not require ethical approval unless mandated by national laws, which was not the case for our research. Accordingly, our study adhered to all relevant ethical and legal standards in the UK, Italy and Belgium.

## Consent

Informed Consent was not obtained because of the retrospective nature of the study.

## Conflicts of Interest

The authors declare no conflicts of interest.

## Supporting information


**Table S1:** Model intercept, coefficients and calibration of the model including estimated fetal weight (EFW) and umbilical artery pulsatility artery (UA PI) discordance.
**Table S2:** Prediction models for stillbirth and/or iatrogenic preterm birth before 34 weeks' gestation for fetal indications (including cases with TTTS). OR, odds ratio; CI, confidence interval; AUC, area under the curve; BMI, body mass index; TTTS, twin to twin transfusion syndrome; EFW, estimated fetal weight; SGA, small for gestational age; UA, umbilical artery; PI, pulsatility index; EDF, end diastolic flow; MCA, middle cerebral artery.
**Table S3:** Prediction models for stillbirth and/or iatrogenic preterm birth before 34 weeks' gestation for fetal indications in women with the last obstetric ultrasound < 34 weeks' gestation and without TTTS (*n* = 201). OR, odds ratio; CI, confidence interval; AUC, area under the curve; BMI, body mass index; EFW, estimated fetal weight; SGA, small for gestational age; UA, umbilical artery; PI, pulsatility index; EDF, end diastolic flow; AR, absent or reverse; MCA, middle cerebral artery.

## Data Availability

The data that support the findings of this study are available from the corresponding author upon reasonable request.
