# Supplementary material for: Prediction of Adverse Perinatal Outcome in Monochorionic Twin Pregnancy Using Fetal Biometry and Doppler Data: A Multicentre Cohort Study
Source: BJOG. 2025 Dec 16;133(4):823–32. doi: 10.1111/1471-0528.70116 (PMC12884219; doi:10.1111/1471-0528.70116)
Supplement: Supplementary file 1 — Table S1: Model intercept, coefficients and calibration of the model including estimated fetal weight (EFW) and umbilical artery pulsatility artery (UA PI) discordance. Table S2: Prediction models for stillbirth and/or iatrogenic preterm birth before 34 weeks' gestation for fetal indications (including cases with TTTS). OR, odds ratio; CI, confidence interval; AUC, area under the curve; BMI, body mass index; TTTS, twin to twin transfusion syndrome; EFW, estimated fetal weight; SGA, small for gestational age; UA, umbilical artery; PI, pulsatility index; EDF, end diastolic flow; MCA, middle cerebral artery. Table S3: Prediction models for stillbirth and/or iatrogenic preterm birth before 34 weeks' gestation for fetal indications in women with the last obstetric ultrasound < 34 weeks' gestation and without TTTS (n = 201). OR, odds ratio; CI, confidence interval; AUC, area under the curve; BMI, body mass index; EFW, estimated fetal weight; SGA, small for gestational age; UA, umbilical artery; PI, pulsatility index; EDF, end diastolic flow; AR, absent or reverse; MCA, middle cerebral artery. [file BJO-133-823-s001.docx]

**Table S1** Model intercept, coefficients and calibration of the model including estimated fetal weight (EFW) and umbilical artery pulsatility artery (UA PI) discordance.

| **Intercept** | **Coefficients** | **Hosmer-Lemeshow Test** |
| --- | --- | --- |
| -4.491 | EFW discordance: 0.097 (p < 0.001) | Chi-square: 10.400 (p = 0.238) |
|  | UA discordance: 0.030 (p < 0.001) |  |

**Table S2** Prediction models for stillbirth and/or iatrogenic preterm birth before 34 weeks’ gestation for fetal indications (including cases with TTTS).

| Models | Variables | OR | 95% CI | AUC | 95% CI |
| --- | --- | --- | --- | --- | --- |
| **Maternal factors** | | | | | |
|  | - Maternal age - BMI - Non-white ethnicity | 1.00  1.05  1.01 | 0.94-1.01  1.01-1.09  0.58-1.77 | 0.59 | 0.53-0.65 |
| **Fetal factors** | | | | | |
| 1 | - TTTS - EFW discordance (%) - UA PI discordance (%) - MCA PI discordance (%) | 5.61  1.07  1.03  1.02 | 2.96-10.65  1.05-1.10  1.01-1.04  1.00-1.04 | 0.86 | 0.81-0.90 |
| 2 | - TTTS - EFW discordance (%) - UA PI discordance (%) | 7.63  1.06  1.03 | 4.51-12.9  1.04-1.08  1.01-1.05 | 0.87 | 0.83-0.91 |
| 3 | - TTTS - SGA<10^th^ centile - UA PI discordance (%) - MCA PI discordance (%) | 6.22  1.58  1.04  1.02 | 3.41-11.35  0.80-3.13  1.02-1.06  1.00-1.03 | 0.79 | 0.73-0.85 |
| 4 | - TTTS - SGA<10^th^ centile - UA PI discordance (%) | 8.50  2.01  1.04 | 5.13-14.09  1.10-3.66  1.03-1.06 | 0.82 | 0.76-0.87 |

OR: odds ratio, CI: confidence interval, AUC: area under the curve, BMI: body mass index, TTTS: twin to twin transfusion syndrome, EFW: estimated fetal weight, SGA: small for gestational age, UA: umbilical artery, PI: pulsatility index, EDF: end diastolic flow, MCA: middle cerebral artery.

**Table S3.** Prediction models for stillbirth and/or iatrogenic preterm birth before 34 weeks’ gestation for fetal indications in women with the last obstetric ultrasound <34 weeks’ gestation and without TTTS (n=201).

| Models | Variables | OR | 95% CI | AUC | 95% CI |
| --- | --- | --- | --- | --- | --- |
| **Maternal factors** | | | | | |
|  | - Maternal age (years) - BMI (kg/m^2^) - Non-white ethnicity | 0.98  1.09  1.56 | 0.92-1.05  1.01-1.17  0.57-4.08 | 0.63 | 0.53-0.72 |
| **Fetal factors** | | | | | |
| 1 | - EFW discordance (%) - UA PI discordance (%) - MCA PI discordance (%) | 1.07  1.02  1.02 | 1.04–1.11  0.99–1.04  0.99–1.04 | 0.76 | 0.68-0.87 |
| 2 | - EFW discordance (%) - UA PI discordance (%) | 1.07  1.03 | 1.04–1.11  1.00–1.05 | 0.80 | 0.73-0.88 |
| 3 | - EFW discordance (%) - Any UA PI AREDF | 1.08  9.04 | 1.05–1.12  2.43–44.57 | 0.82 | 0.74-0.89 |
| 4 | - SGA<10th centile - UA PI discordance (%) - MCA PI discordance (%) | 2.01  1.03  1.01 | 0.78–5.96  1.01–1.05  0.99–1.04 | 0.65 | 0.55-0.75 |
| 5 | - SGA<10th centile - UA PI discordance (%) | 2.04  1.04 | 0.86–5.43  1.02–1.06 | 0.69 | 0.60-0.78 |
| 6 | - SGA<10th centile - Any UA PI AREDF | 2.51  13.33 | 1.07–6.66  4.00–60.75 | 0.68 | 0.61-0.75 |

OR: odds ratio, CI: confidence interval, AUC: area under the curve, BMI: body mass index, EFW: estimated fetal weight, SGA: small for gestational age, UA: umbilical artery, PI: pulsatility index, EDF: end diastolic flow, AR: absent or reverse, MCA: middle cerebral artery.
